# Molecular transition of SARS-CoV-2 from critical patients during the first year of the COVID-19 pandemic in Mexico City

**DOI:** 10.3389/fcimb.2023.1155938

**Published:** 2023-05-16

**Authors:** Aldo Hugo De La Cruz-Montoya, Clara Estela Díaz Velásquez, Héctor Martínez-Gregorio, Miguel Ruiz-De La Cruz, José Bustos-Arriaga, Tannya Karen Castro-Jiménez, Jonadab Efraín Olguín-Hernández, Miriam Rodríguez-Sosa, Luis Ignacio Terrazas-Valdes, Luis Armando Jiménez-Alvarez, Nora Elemi Regino-Zamarripa, Gustavo Ramírez-Martínez, Alfredo Cruz-Lagunas, Irlanda Peralta-Arrieta, Leonel Armas-López, Belinda Maricela Contreras-Garza, Gabriel Palma-Cortés, Carlos Cabello-Gutierrez, Renata Báez-Saldaña, Joaquín Zúñiga, Federico Ávila-Moreno, Felipe Vaca-Paniagua

**Affiliations:** ^1^Laboratorio Nacional en Salud, Diagnóstico Molecular y Efecto Ambiental en Enfermedades Crónico-Degenerativas, Facultad de Estudios Superiores Iztacala, Tlalnepantla, Mexico; ^2^Unidad de Investigación en Biomedicina, Facultad de Estudios Superiores Iztacala, Universidad Nacional Autónoma de México, Tlalnepantla, Mexico; ^3^Departamento de Infectómica y Patogénesis Molecular, Centro de Investigación y de Estudios Avanzados del Instituto Politécnico Nacional (CINVESTAV-IPN), Avenida Instituto Politécnico Nacional, Colonia San Pedro Zacatenco, Delegación Gustavo A. Madero, Ciudad de México, Mexico; ^4^Laboratorio de Inmunobiología y Genética y Departamento de Virología, Instituto Nacional de Enfermedades Respiratorias (INER) Ismael Cosio Villegas, Ciudad de México, Mexico; ^5^Tecnológico de Monterrey, Escuela de Medicina y Ciencias de la Salud, Ciudad de México, Mexico; ^6^Instituto Nacional de Enfermedades Respiratorias (INER) Ismael Cosio Villegas, Ciudad de México, Mexico; ^7^Department of Research in Virology and Mycology, Instituto Nacional de Enfermedades Respiratorias (INER) Ismael Cosio Villegas, Ciudad de México, Mexico; ^8^Laboratorio 12 de Enfermedades Pulmonares y Epigenómica del Cáncer, Unidad de Investigación en Biomedicina (UBIMED), Facultad de Estudios Superiores Iztacala, Universidad Nacional Autónoma de México, Tlalnepantla, Mexico; ^9^Subdirección de Investigación Básica, Instituto Nacional de Cancerología, Ciudad de México, Mexico

**Keywords:** SARS-CoV-2, genome, COVID-19, mutational signatures, evolutionary forces

## Abstract

**Background:**

The SARS-CoV-2 virus has caused unprecedented mortality since its emergence in late 2019. The continuous evolution of the viral genome through the concerted action of mutational forces has produced distinct variants that became dominant, challenging human immunity and vaccine development.

**Aim and methods:**

In this work, through an integrative genomic approach, we describe the molecular transition of SARS-CoV-2 by analyzing the viral whole genome sequences from 50 critical COVID-19 patients recruited during the first year of the pandemic in Mexico City.

**Results:**

Our results revealed differential levels of the evolutionary forces across the genome and specific mutational processes that have shaped the first two epidemiological waves of the pandemic in Mexico. Through phylogenetic analyses, we observed a genomic transition in the circulating SARS-CoV-2 genomes from several lineages prevalent in the first wave to a dominance of the B.1.1.519 variant (defined by T478K, P681H, and T732A mutations in the spike protein) in the second wave.

**Conclusion:**

This work contributes to a better understanding of the evolutionary dynamics and selective pressures that act at the genomic level, the prediction of more accurate variants of clinical significance, and a better comprehension of the molecular mechanisms driving the evolution of SARS-CoV-2 to improve vaccine and drug development.

## Introduction

Severe acute respiratory syndrome coronavirus 2 (SARS-CoV-2) was identified as the causal agent of pneumonia in a cluster of patients in Wuhan, China in late December 2019 and led off the pandemic. SARS-CoV-2 is an enveloped virus with a linear positive-sense single-stranded RNA genome that belongs to the *Coronaviridae* family and causes the infectious coronavirus disease 2019 (COVID-19) (Zhu et al., 2020). Its genome is composed of 29,903 bases distributed across several open reading frames (ORF), starting with a 5’-untranslated region (5’-UTR) followed by two-overlapping ORFs that comprise almost three-quarters of the genome, composed by ORF1a and ORF1b which code for 16 nonstructural proteins (Nsp) that take part in viral replication and transcription. Then, various sequences that are expressed as subgenomic RNAs that code for accessory proteins such as ORF3a, ORF6, ORF7a, ORF7b, ORF8, and ORF10, four coding for structural proteins S, E, M and N, all flanked by a 3’-UTR (Michel et al., 2020; Naqvi et al., 2020).

Mexico has experienced five COVID-19 waves, with a total of 7,541,591 confirmed cases and 333,531 deaths as to April 3, 2023 (Worldometers.info, 2023). During the first year, in low and middle-income countries, factors such as poverty, access to healthcare, and increased population risk of several metabolic and cardiovascular diseases made the pandemic particularly difficult to handle. This was reflected in a higher mortality rate due to COVID-19 in Latin America, specifically in countries like Mexico where 1,992,794 cases and 196,525 deaths were reported only in the first year (Worldometers.info, 2023), compared with high-income countries like those from western Europe (Ortiz-Hernández and Pérez-Sastré, 2020; Palacio-Mejía et al., 2022).

SARS-CoV-2 has a high replication fidelity mainly dependent on the RNA-dependent on the 3’ to 5’ exoribonuclease activity of the nonstructural protein 14 (Nsp14) and therefore a low mutation rate compared with other viruses (Denison et al., 2011). Nsp14 has been associated with proofreading activity, reducing the mutation rate in the viral genome during its replication (Ma et al., 2015). Even so, many mutations have been identified throughout the SARS-CoV-2 genomes sequenced so far, which means that there are other mechanisms such as oxidative stress and RNA editing which could be shaping the SARS-CoV-2 mutational profile (Di Giorgio et al., 2020; Simmonds, 2020; Graudenzi et al., 2021; Graudenzi et al., 2021; Mourier et al., 2021; Ratcliff and Simmonds, 2021).

The first SARS-CoV-2 genome reported from a Mexican patient was published in May 2020 and belonged to the subclade A2a, lineage G (Garcés-Ayala et al., 2020). An initial genomic analysis from 17 whole genome SARS-CoV-2 sequences isolated from Mexican patients reported the presence of the H49Y aa substitution in the Spike protein and the phylogenetic analysis showed that the viruses sequenced belonged to the A2/G and B/S lineages, two of the three lineages circulating at that time (Taboada et al., 2020). The rapid availability of vast SARS-CoV-2 genomic data has been a key factor in the response to the pandemic; from tracking, diagnosing, and following transmission across populations to the design and development of vaccines and antivirals (Amanat and Krammer, 2020; Zhang and Holmes, 2020; Dellicour et al., 2021). The study of these genomic data is crucial to understand the selective pressures and the evolutionary dynamics acting on the SARS-CoV-2 genome, predicting more accurate variants of clinical significance, better understanding the molecular mechanisms driving SARS-CoV-2 evolution, and improving vaccine and drug development.

In this study, we extensively describe the molecular transition of SARS-CoV-2 from critical COVID-19 patients from the first year of the pandemic in Mexico City through an integrative genomic approach.

## Materials and methods

### Population of study

**Figure 1:**
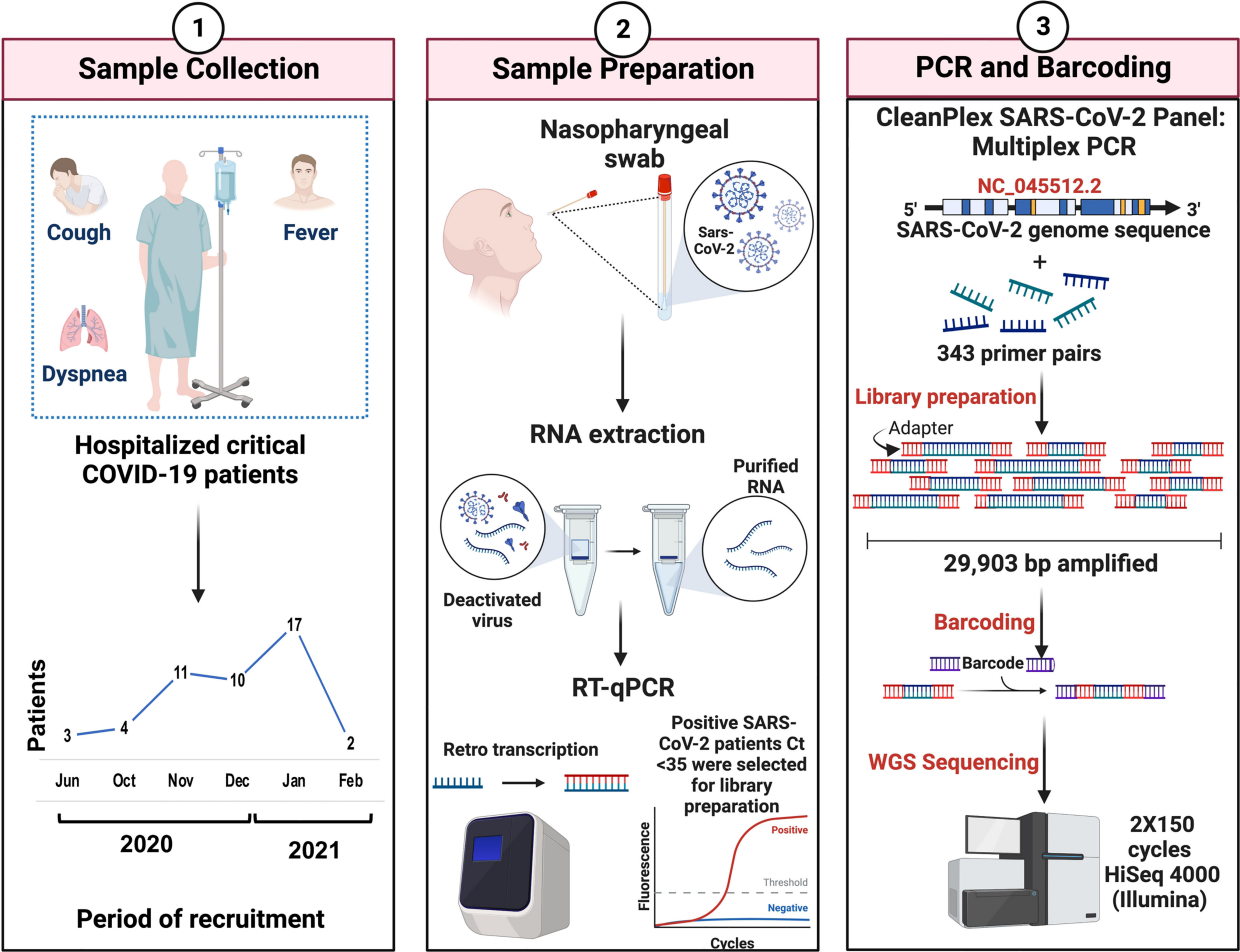
Overview of the study workflow. Critical COVID-19 patients were selected according to the COVID-19 Treatment Guidelines (Panel 1). Nasopharyngeal samples were collected, and the viral RNA extraction of SARS-CoV-2-positive patients was carried out. Criteria of <35 ct in RT-qPCR were required for the preparation of genomic libraries (Panel 2). Library preparation was performed using multiplex PCR and WGS was performed in a 2X150 cycles format on an Illumina HiSeq (Panel 3).

Nasopharyngeal swab samples were obtained from 200 patients from the National Institute of Respiratory Diseases (INER) in Mexico City from June 2020 to February 2021 (**Figure 1**, panel 1), within the approved protocols B28-16 and B09-20. Patients were grouped into mild, severe, and critical according to the COVID-19 Treatment Guidelines. The National Institutes of Health guidelines for treatment categorize mild COVID as patients with symptoms such as fever, cough, sore throat, malaise, headache, muscle pain, nausea, vomiting, diarrhea, and loss of taste and smell. According to the Infectious Diseases Society of America, severe COVID is defined by SpO2 ≤94% on room air, including patients on supplemental oxygen and symptoms like shortness of breath, dyspnea, and abnormal chest imaging. Critical COVID is characterized by the presence of symptoms previously mentioned and often the need for mechanical ventilation and extracorporeal membrane oxygenation (ECMO), and the existence of some of the following: respiratory failure, septic shock, and/or multiple organ dysfunction (Chan, 2022).

### RNA extraction and sample selection

Total RNA was extracted using the QIAmp Viral RNA Mini kit (QIAGEN, Hilden, Germany) in the presence of 5 µg/mL of carrier RNA and quantified by fluorometry in a Quantus Fluorometer (Promega Corporation, Madison, Wisconsin, USA). Fifty nanograms from each sample were analyzed by qPCR using the GeneFinder COVID-19 Plus RealAmp Kit (OSANG Healthcare Co., Ltd, South Korea) and all samples with a Ct <35 were selected for library preparation (**Figure 1**, panel 2).

### Library preparation and SARS-CoV-2 whole genome sequencing

Library preparation was performed employing the CleanPlex SARS-CoV-2 Research and Surveillance Panel (Paragon Genomics, Inc., Hayward, California, USA) according to the manufacturer’s instructions. Briefly, total RNA was used for cDNA synthesis and purification. Then, a multiplex PCR with target-specific primers to amplify targets of interest was carried out, covering the entire SARS-CoV-2 genome with a 2-pool design, followed by a digestion reaction that achieves background cleaning by removing nonspecific PCR products. Finally, a PCR reaction with CleanPlex Indexed PCR Primers to amplify and add indexes to the generated libraries was conducted. After library preparation, the amplification of viral RNA was verified by automated electrophoresis using a 2100 Bioanalyzer (Agilent Technologies, Inc., Santa Clara, California, USA). The presence of a peak at ~275 bp indicated the successful amplification of the targeted regions. The samples were normalized by molar concentration, pooled, and sequenced for 2X150 cycles in a HiSeq 4000 (Illumina, San Diego, California, USA) at Novogene (Sacramento, California, USA) (**Figure 1**, panel 3).

### Bioinformatic analysis

SOPHiA DDM software (SOPHiA Genetics Inc., Boston, Massachusetts, USA) was used to align reads to the reference genome (NC_045512.2), read filtering, and variant calling. First, read mapping adaptors were trimmed, mispriming events were eliminated, read softclipped regions were realigned and primer sequences trimmed. Reads shorter than 21 bp were excluded, and the resulting alignment was used for variant calling using SOPHiA DDM pipeline. FASTA and annotated files were downloaded for downstream analysis. Mutations were considered from samples with a coverage of at least 98% and a mutation fraction of at least 70% (Kubik et al., 2021). To visualize the molecular transition of SARS-CoV-2 mutations were plotted and segmented by year using the ComplexHeatmap R package (Gu et al., 2016).

### Molecular characterization and evolutionary analysis

For the identification of molecular characteristics of SARS-CoV-2, mutations from 50 critical patients were classified as single nucleotide variants (SNV), synonymous, and nonsynonymous. Mutations were analyzed by genomic region, normalized to each region length and plotted in the whole genome using R. The overall transition/transversion ratio was calculated with MEGA software V.11.0 (Tamura et al., 2021).

579 complete SARS-CoV-2 genome sequences (>29,000 nt) with high coverage uploaded from Mexico City to the Global Initiative on Sharing Avian Influenza Data (GISAID) database within 2020-06-01 to 2021-02-28 were selected. FASTA files were downloaded to study the evolutionary forces acting on SARS-CoV-2 genomes along with the 50 whole genome sequences from this study. Using these 629 sequences, we evaluated the transition/transversion ration for each genomic region and assessed neutrality with Tajima’s D test using DNASP6 software (Rozas et al., 2017) and positive selection with Datamonkey tool (Weaver et al., 2018).

### Mutational signatures

For the mutation signature analysis, we used Alexandrov´s strategy (Alexandrov et al., 2013). We evaluated all changes in a trinucleotide context where the mutated nucleotide is in the center. In this approach, there are 192 (12*16) possible changes. For the ZAP mutational signature, we used a dinucleotide context because this enzyme acts in CpG nucleotide pairs (16 different dinucleotide combinations). We used a 12- and 6-substitution pattern to present these data (Graudenzi et al., 2021). Then, we analyzed single nucleotide changes in every SARS-CoV-2 region and normalized them to region length to identify specific mutational signatures in each region. All mutational signatures were plotted in R using ggplot2 package (Villanueva and Chen, 2019).

### Protein modeling

PyMOL (DeLano, 2002) was used to model the SARS-CoV-2 spike glycoprotein and identify the mutated sites in our samples. The structure of the spike protein (id:7DZW) was downloaded from the Protein Data Bank (PDB) (Kouranov et al., 2006) and modeled in two ways: i) using all the mutations identified in our cohort and ii) using only the mutations identified in the January-February 2021 period.

### Phylogenetic analysis

Phylogenetic trees were constructed using Nextstrain and visualized with Auspice v2.37.3. The tool Nexctlade v.2.5.0 was used to compare the 50 whole-genome sequences to SARS-CoV-2 reference sequences and determine their location in the phylogenetic tree (Hadfield et al., 2018).

## Results

### Patient characteristics

**Figure 2:**
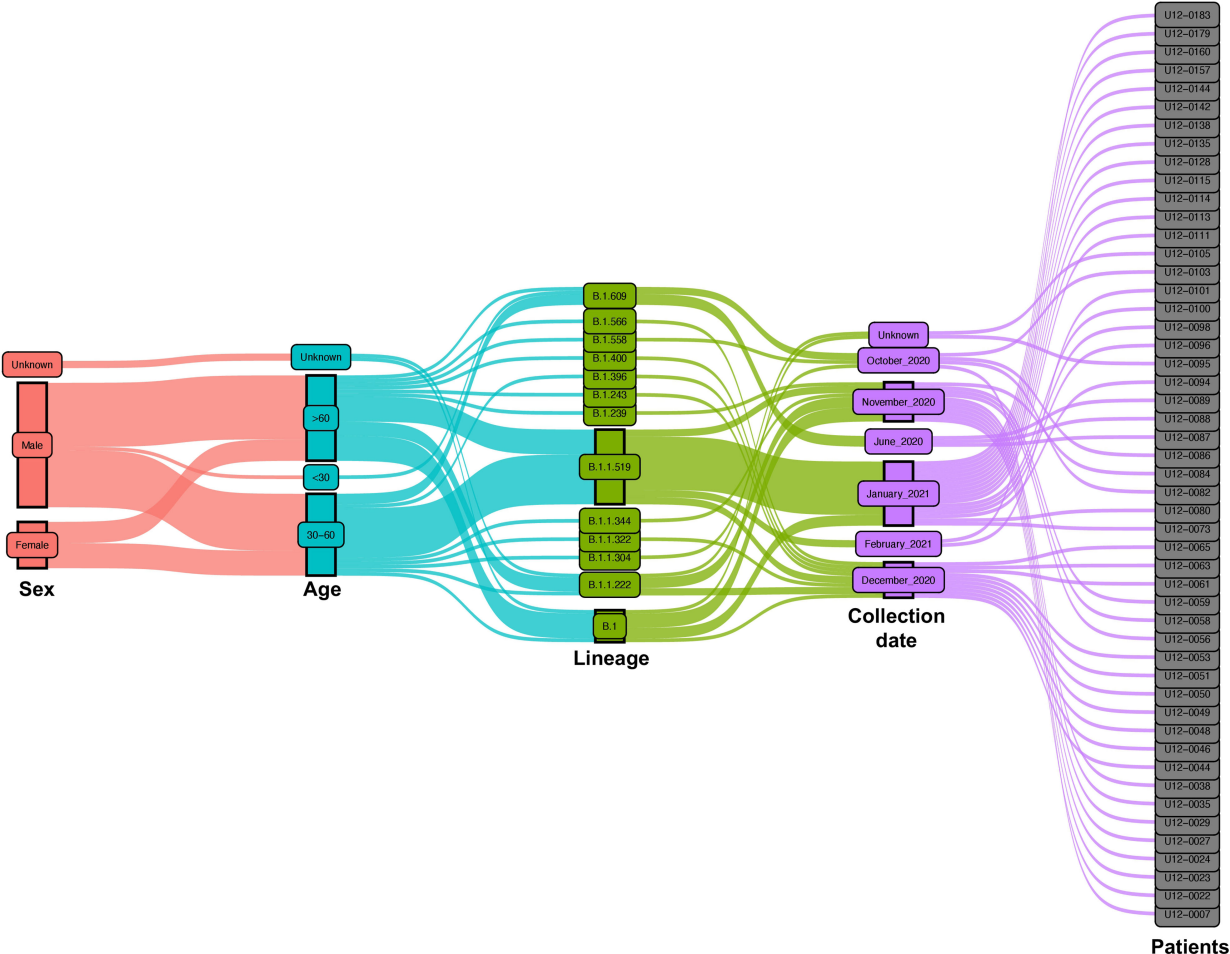
Epidemiological and virological characteristics of critical COVID-19 Mexican patients. Demographic parameters, nasopharyngeal swab collection dates, and virologic parameters of the patients are represented in an alluvial diagram, each one depicted in a different color. The distribution of the main lineages of SARS-CoV-2 is sketched during the 8-month study period from June 2020 to February 2021.

For this study, we collected nasopharyngeal swab samples over eight months (June 2020 to February 2021) from a total of 200 COVID-19-positive patients. After qPCR analysis only samples with a Ct <35 were selected for library preparation and whole genome sequencing (**Figure 1**). After this filter, 50 patients were selected from which 35 (70%) are men and 13 (26%) are women. Most of them were older than 30 years with mean ages of 59.1 and 57.2, respectively. Only 4 patients were diagnosed with mild disease (8%) and the other 46 patients were diagnosed with critical disease (92%) (**Figure 2**).

### Global mutational analysis of SARS-CoV-2

**Figure 3:**
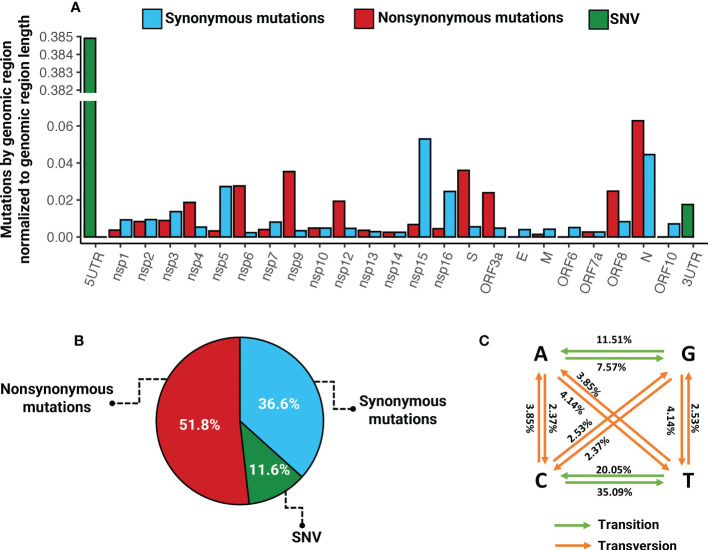
Mutational profile of SARS-CoV-2 in critical COVID-19 patients. **(A)** Distribution of nonsynonymous, synonymous mutations, and single nucleotide variants across the SARS-CoV-2 genome. **(B)** Percentage of nonsynonymous, synonymous mutations, and single nucleotide variants. **(C)** Percentage of transitions and transversions.

From the 50 SARS-CoV-2 whole genome sequences, we identified a total of 923 mutations (mean = 18.46, range 5 - 28) across 12 different ORFs, 5’, and 3’-UTRs. Of these mutations, 48.2% were synonymous and 51.8% were nonsynonymous (**Figure 3B**). The highest percentage of synonymous mutations are present in the 5’ untranslated region (UTR) and sequences that code for Nsp3 and Nsp15, ORF1a and ORF1b, respectively. While the S, ORF1b, and N regions had the largest number of nonsynonymous mutations distributed across them (**Figure 3A**). Among the sequences that code for structural proteins, the two with more mutations were S and N. In the S region we identified 159 nonsynonymous (17.22%) and 21 synonymous mutations (2.27%), while in N we identified 79 nonsynonymous (8.55%) and 56 synonymous mutations (6.06%) (**Figure 3A**). We performed a mutational analysis and found diverse mutational transition and transversion patterns dominated by a majority of C>T (35.09%), followed by T>C (20.05%), G>A (11.51%), and A>G (7.57%) transitions (**Figure 3C**).

### Evolutionary analysis of SARS-CoV-2

To evaluate positive selection across the whole viral genome, we calculated the dN/dS (nonsynonymous/synonymous ratio) from 629 complete SARS-CoV-2 genomic sequences. Four amino acid changes showed significant positive selection: P141S (Nsp3), T492I (Nsp4), P681H (S), and T732A (S) (**Table 1**). In contrast, when we evaluated Tajima’s D test, there was a tendency for purifying selection in all genomic regions. Ti/Tv showed higher values in Nsp4, Nsp5, Nsp9, Nsp10, Nsp15, and ORF6 (**Table 2**).

**Table 1:** Nonsynonymous/Synonymous mutation ratio from 629 complete SARS-CoV-2 genome sequences from Mexico in the period from June 2020 to February 2021.

Nucleotide change	Amino Acid change	Genomic location	dN/dS (p-value)
C421T	P141S	ORF1a (Nsp3)	7.00 (*p*=0.003)
C1475T	T492I	ORF1a (Nsp4)	3.00 (*p*=0.088)
C2042A	P681H	S	3.34 (*p*=0.082)
A2194G	T732A	S	6.00 (*p*=0.039)

**Table 2:** Transition/transversion ratio and Tajima’s D neutrality test per genomic region from 629 complete SARS-CoV-2 genome sequences from Mexico in the period from June 2020 to February 2021.

Genomic location	Tajima's D	Ti/Tv
Nsp1	-2.6	6.3
Nsp2	-2.8	2.4
Nsp3	-2.8	3.5
Nsp4	-2.7	13.9
Nsp5	-2.5	19.4
Nsp6	-2.5	5.0
Nsp7	-2.3	7.2
Nsp8	-2.5	7.2
Nsp9	-2.0	44.0
Nsp10	-2.3	13.8
Nsp12	-2.8	2.3
Nsp13	-2.7	3.7
Nsp14	-2.8	3.1
Nsp15	-2.5	12.0
Nsp16	-2.5	9.1
S	-2.8	1.1
ORF3a	-2.7	1.3
E	-2.2	1.0
M	-2.6	1.5
ORF6	-2.2	11.7
ORF7a	-2.6	4.1
ORF8	-2.6	2.3
N	-2.5	2.4
ORF10	-2.1	4.9

### Mutational signature analysis

**Figure 4:**
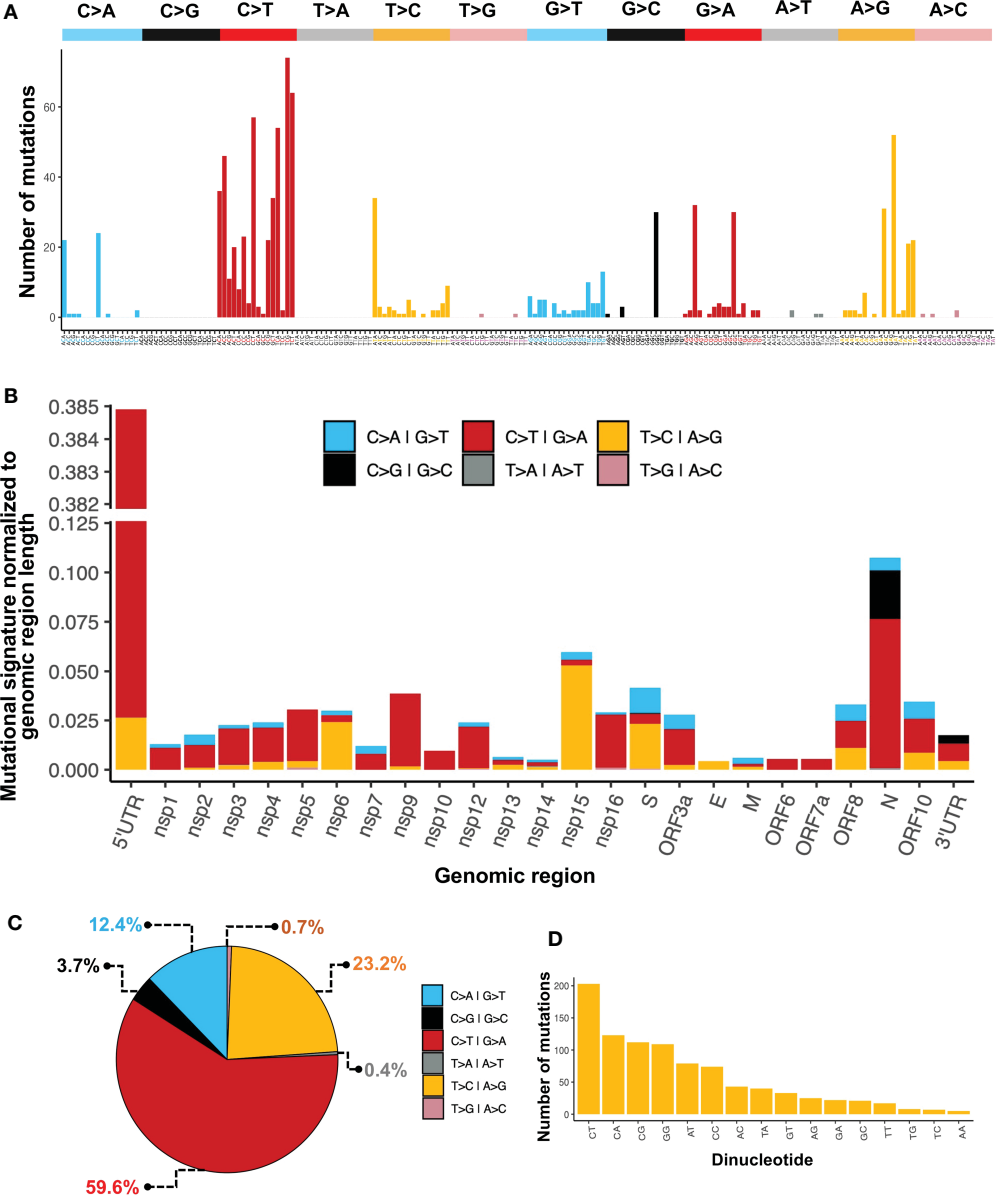
Mutational patterns identified in the SARS-CoV-2 genome. **(A)** Mutational signature analysis in the context of three nucleotides. **(B)** Mutational signature analysis in a one-nucleotide context normalized to region length. **(C)** Percentage of one-nucleotide changes. **(D)** Mutational signature analysis in a dinucleotide context.

We performed a mutational signature analysis in the context of one, two, and three nucleotides. In a one-nucleotide context, we identified that most changes were C>T/G>A (59.6%), followed by T>C/A>G (23.2%), and C>A/G>T (12.4%) (**Figure 4C**). Then, we normalized the number of mutations to sequence length to determine the mutational signature distribution along the SARS-CoV-2 genome. We found that most C>T/G>A changes were overrepresented mainly in the 5’UTR, followed by the N region. While the T>C/A>G changes were mainly found in the Nsp15 followed by Nsp6, and S. Although C>G/G>C changes were not the most prevalent across the genome, these changes were found distributed only between N and the 3’UTR (**Figure 4B**). We also identified that the most common single nucleotide changes in the context of three nucleotides were those of C>T followed by A>G (**Figure 4A**). Finally, in the dinucleotide context, where the first nucleotide is the mutation and the second is the next nucleotide in the 5’>3’ direction, the most prevalent dinucleotide changes were CpT, CpA, CpG, and GpG (**Figure 4D**).

### Genomic landscape of SARS-CoV-2

**Figure 5:**
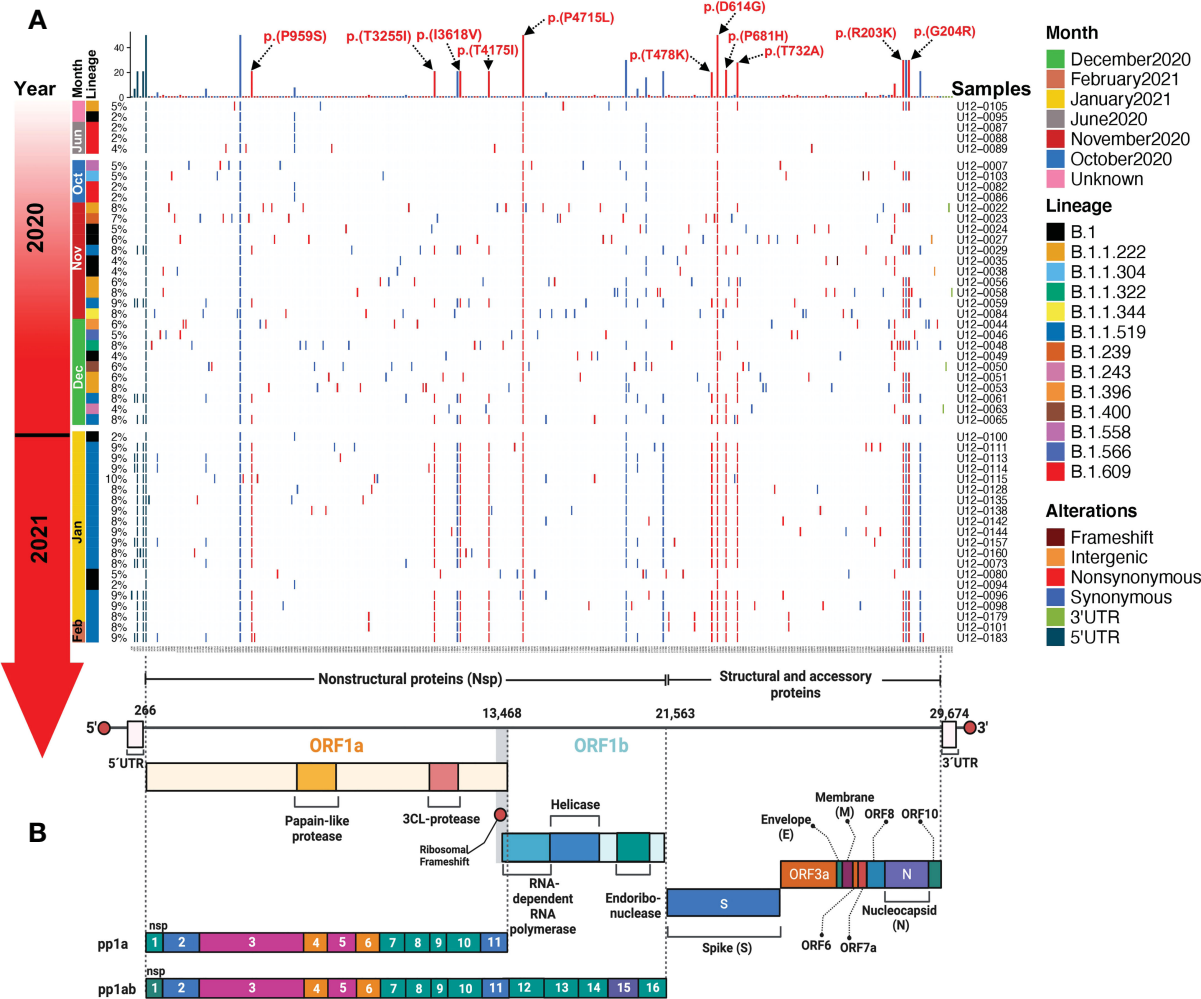
Landscape of the SARS-CoV-2 mutational transition from critical Mexican patients during the first year of the COVID-19 pandemic in Mexico. **(A)** Mutations identified across SARS-CoV-2 genomes are represented in a complex heatmap. **(B)** Architecture of the SARS-CoV-2 RNA genome; nonstructural, structural, and accessory proteins are indicated. Red lines represent nonsynonymous mutations, blue lines represent synonymous mutations, light green lines represent 3’ UTR mutations, and dark green lines represent 5’ UTR mutations.

The S:D614G and ORF1b:P323L amino acid (aa) substitutions in the spike protein and the RNA-dependent RNA polymerase (RdRp), respectively, were present in all 50 SARS-CoV-2 genomes, co-occurring with two synonymous mutations; C241T in the 5’-UTR and C3037T in the Nsp3. Other frequent aa changes in S across samples were T732A (28%), P681H (22%), and T478K (20%). These three aa substitutions in the S sequence define the B.1.1.519 Phylogenetic Assignment of Named Global Outbreak (PANGO) lineage, which we found in 21 (42%) of our samples, followed by B.1 (9/50, 18%), B.1.1.222 (6/50, 12%), and B.1.609 (5/50,10%). More aa substitutions that characterized the B.1.1.519 lineage that were present in our samples are P959S, T3255I, I3618V, and T4175I in ORF1a and R203K, and G204R in N (**Figure 5A, B**).

### Protein alterations by modeling analysis

**Figure 6:**
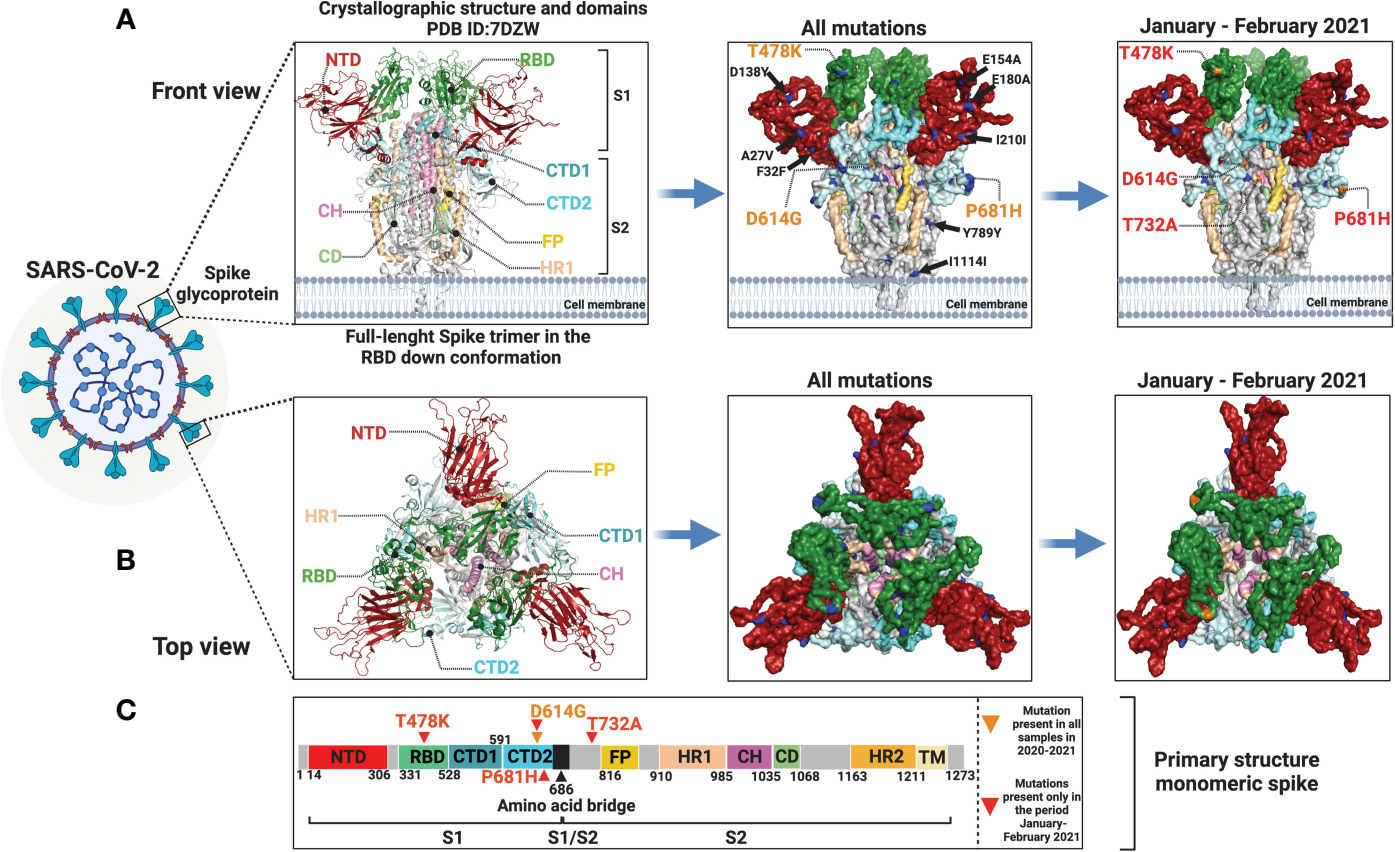
Protein modeling of SARS-CoV-2 spike glycoprotein mutation distribution. Crystallographic structures of spike protein are depicted in a **(A)** front view (left panel) and in a **(B)** top view (left panel). In the middle panel **(A, B)**, all mutations identified in this study are represented by blue and orange dots and orange letters. In the right panel **(A, B)**, only mutations identified in the January-February 2021 period are shown in blue and orange dots and red letters. Mutations with low allelic frequency are presented in black letters. **(C)** Distribution of amino acid changes in the primary structure of spike protein.

We modeled the SARS-CoV-2 spike glycoprotein structure and identified the mutated sites represented in our samples. In the left panel of **Figure 6**, we can observe the location of the different structural domains along the spike protein. In the middle panel, all the mutations identified in our cohort in the whole study are shown and the D614G amino acid substitution which is present in all samples is indicated. In the right panel, only the mutations identified in the January-February 2021 period are represented. The distribution of amino acid changes represented in the primary structure of the SARS-CoV-2 spike protein is depicted in **Figure 6C**.

### Phylogenetic analysis

**Figure 7:**
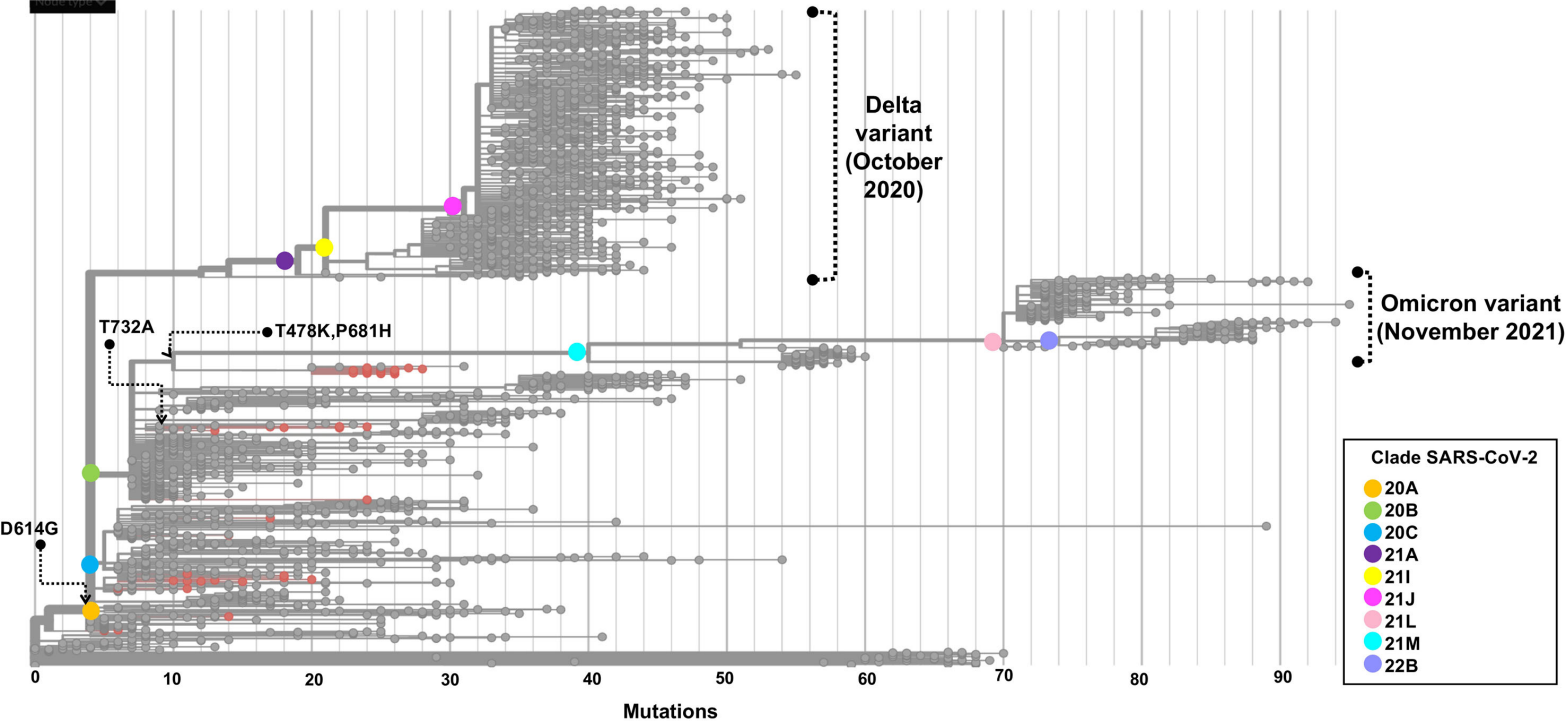
Phylogenetic tree of a SARS-CoV-2 strains from COVID-19 critical patients in Mexico City. The root of the phylogenetic tree is the reference genome isolated in Wuhan-Hu-1 (NC_045512). Strains are distributed by clade, and samples sequenced in this study are represented by red dots. The set of these mutations precedes the origin of the omicron variant. The phylogenetic tree was generated and modified for display purposes from Nextstrain (https://nextstrain.org/ncov). Emergence dates from Delta and Omicron variants are shown.

The phylogenetic tree depicted in **Figure 7** shows the phylogenetic analysis of 50 whole-genome SARS-CoV-2 sequences from critical COVID-19 patients circulating during the first two waves in Mexico. The samples are grouped through clades 20A, 20C, and 20B, in ascending order regarding the number of mutations compared with the reference genome.

## Discussion

**Figure 8:**
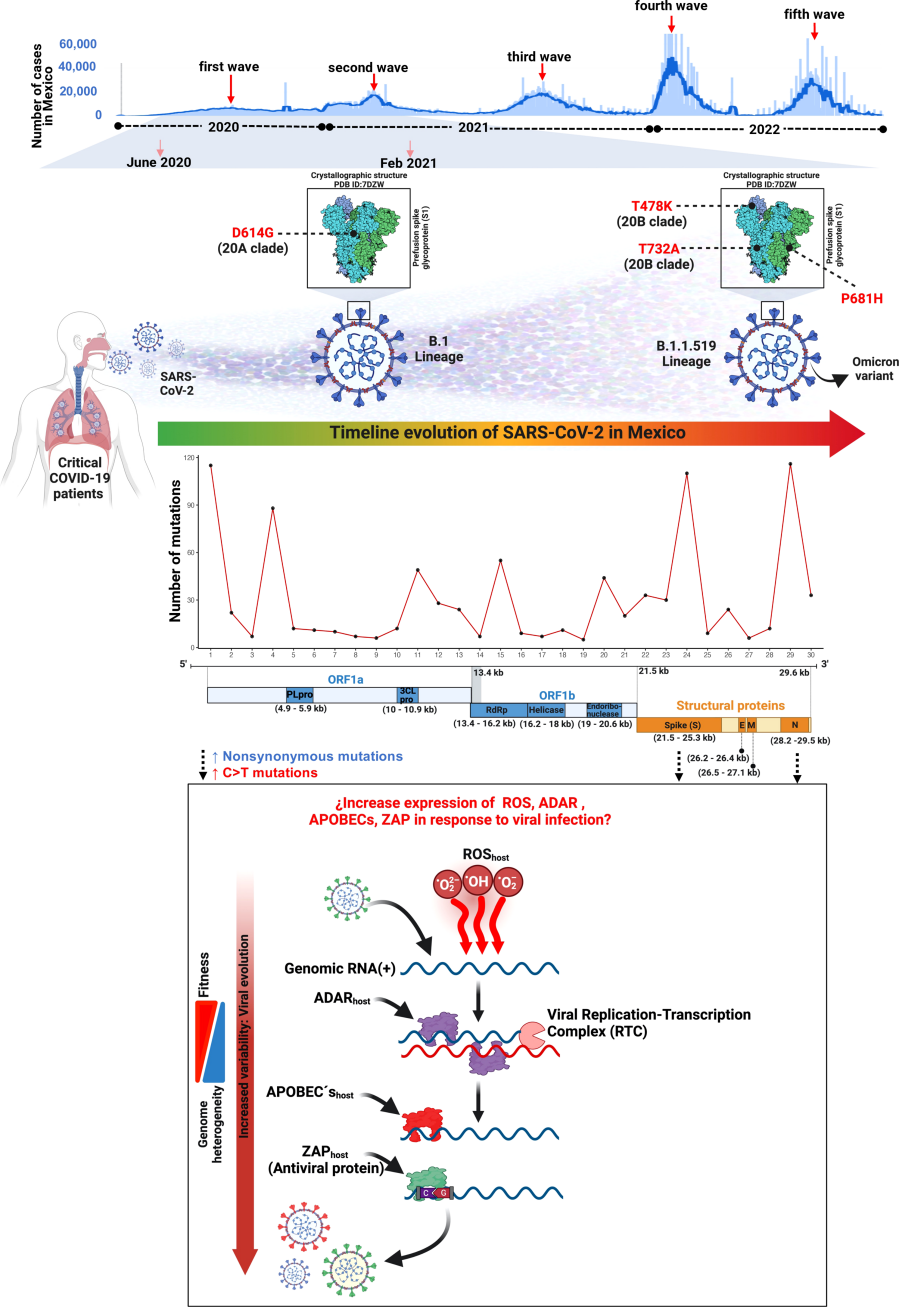
Landscape of the SARS-CoV-2 molecular transition from critical patients during the first year of the COVID-19 pandemic in Mexico.

In this work, we reported the genomic landscape of whole-genome sequences from critical COVID-19 patients within the first and second COVID-19 waves during the first year of the pandemic in Mexico City (**Figure 8**).

We recruited 200 COVID-19 patients from January 2020 to February 2021 and collected nasopharyngeal swab samples. All samples were evaluated by qPCR, and 50 samples that passed a Ct <35 quality filter were selected for library preparation and whole genome sequencing. Most patients in this study were men (70%), and older than 30 years (mean age=58.1), comparable to previous works performed in Mexican (Taboada et al., 2021) and other populations (Jin et al., 2020). Most critical patients (40%) harbored the B.1.1.519 variant. Unfortunately, and due to privacy matters, in this study we were not able to collect clinical characteristics. However, it has been reported that patients harboring the B.1.1.519 variant showed an increase in respiratory symptoms unlike those harboring other variants. Additionally, in previous studies, logistic regression showed an association with increased dyspnea, chest pain, and cyanosis (Cedro-Tanda et al., 2021), which could explain the critical condition of most patients in this work.

Mutations in the SARS-CoV-2 genome can be neutral or deleterious, and some mutations provide evolutionary advantages at the molecular level, and may be associated with enhanced fitness, increased infectivity, and immune evasion (Korber et al., 2020; Li et al., 2021; Plante et al., 2021). To understand the evolution of the SARS-CoV-2 genome, we assessed the evolutionary forces that act on its loci. Overall, we found a higher proportion of nonsynonymous than synonymous mutations, 51.8% versus 48.2%, respectively. Some regions, such as Nsp6, Nsp9, Nsp12, S, ORF3a, ORF8, and N exhibited more nonsynonymous than synonymous mutations. On the other hand, we detected a higher rate of transversion than transitions in all the genome, however, transversion was not detected in some regions such as Nsp9, Nsp10, E, ORF6, and ORF7a.

To evaluate the neutrality and a positive selection in SARS-CoV-2 we performed Tajima’s D test and calculated the dN/dS ratio. Neutrality analysis of Tajima’s D showed that all genomic regions are under purifying selection. On the other hand, P141S (Nsp3), T492I (Nsp4), P681H (S), and T732A (S) showed significance in the dN/dS ratio, indicating that they are under positive selection. In addition, our results show that some regions with a high rate of nonsynonymous mutations are not positively selected. These data suggest that different selection pressures may act together on the evolution of SARS-CoV-2 improving fitness or establishing purifying selection.

Notably, the higher rate of nonsynonymous mutations in Nsp12, Nsp4, Nsp6, Nsp3, and Nsp9, where all key components of the replication machinery of the virus relay, may impact the replication efficiency, the acquisition of novel mutation, and fitness. Nsp12 is the N-terminal region of the RNA-dependent RNA polymerase; missense mutations in this region of the genome could influence the mutation rate of the polymerase (Hillen et al., 2020). Nsp4 and Nsp6 are predicted to participate in the anchoring of the replication complex to double membrane vesicles in the cytoplasm (Prentice et al., 2004). Nsp3 interacts with other Nsp proteins and with the viral RNA to form the replication complex (Ziebuhr et al., 2000). Nsp9 is an RNA-binding protein that has been proposed to participate in the replication complex (Egloff et al., 2004). Therefore, the aggregation of mutations in the essential regions of viral replication may facilitate adaptability and transmission by accelerating mutation acquisition (Yao et al., 2020). We also identified several mutations in the 5’ and 3’ UTRs. It is known that there are implications of the genetic variability in these regions that could impact the stability of predicted secondary structures that have been proposed to regulate the translation of viral proteins and compactness of the viral genome of other viruses and are molecular targets of antiviral candidates (Tubiana et al., 2015; Zhao et al., 2020; Miao et al., 2021).

Molecular mimicry is an effective evolutionary adaptation for viral infections to evade immune response mechanisms (Lucas et al., 2008; Strumillo et al., 2021). Most genomic SARS-CoV-2 studies have focused on the analysis of nonsynonymous mutations. However, it has been reported that synonymous changes account for approximately 30% of all SARS-CoV-2 mutations. As a result of genomic large-scale analysis, it has been shown that synonymous mutations that increase the similarity of viral codons to human codons are more likely to become fixed in the SARS-CoV-2 genome throughout time, thereby adapting to the human host and improving the usage of the human aminoacyl-tRNA set (Ramazzotti et al., 2022).

The SARS-CoV-2 mutational signatures of our study revealed that C>T, T>C, and C>A are the predominant single-base substitutions. This mutational asymmetry phenomenon has been observed in SARS-CoV and SARS-CoV-2 (Simmonds, 2020). In addition, the RNA viruses HCV, FMDV, MNV, HPgV-1, and rubella virus have shown an excess of C>T changes over the reverse (T>C) and complementary (G>A) transitions (Simmonds and Ansari, 2021).

The mutational processes and the subjacent mechanisms that seem to be shaping the SARS-CoV-2 genome through an increment in the C>T change frequency in RNA viruses remain to be elucidated, although there is previous evidence that supports three main mutational mechanisms that may cause mutational asymmetry: i) RNA transcription, ii) reactive oxidative species (ROS), and iii) RNA editing (Simmonds, 2020) (**Figure 8**).

RNA transcription is an important source of mutations due to transcription errors introduced by polymerases during the replication of RNA viruses. Nevertheless, biochemical characterizations of poliovirus RNA-dependent RNA polymerase (RdRp) and HIV reverse transcriptase did not identify an increase in C>T mutations over other changes (Ward et al., 1988; Kati et al., 1992; Freistadt et al., 2007). Therefore, the quantitative role of RNA transcription in SARS-CoV-2 mutation accumulation requires further studies, particularly in the context of the pandemic situation.

Oxidative stress inside the cells is another proposed mutational process of the SARS-CoV-2 genome. Recent work suggests that G>T transversions across the SARS-CoV-2 genome might be the consequence of RNA mutational damage in periods of oxidative stress (Graudenzi et al., 2021; Mourier et al., 2021). Reactive oxidative species (ROS) are broadly associated with DNA mutations that produce 8-oxoguanine from guanines. 8-oxoguanine bases do not block DNA synthesis and are copied as adenines, resulting in G>T changes (David et al., 2007). ROS are known to target single-stranded RNA (Li et al., 2006) and their production during viral infections could generate G>T transversions in SARS-CoV-2 genomes. Consistent with our findings, there is evidence that supports higher frequencies of G>T than T>G or C>A changes are present in coronaviruses rather than other RNA viruses (Simmonds, 2020).

A third mutational process affecting the SARS-CoV-2 genome can be RNA editing. During replication, RNA viruses are susceptible to acquiring mutations due to RNA editing antiviral mechanisms in vertebrate cells. Among the most studied mechanisms are adenosine deaminase acting on RNA type 1 (ADAR1) and the apolipoprotein B mRNA editing enzyme catalytic polypeptide (APOBEC) family of enzymes. ADAR1 catalyzes adenine deamination to form inosine which successively is copied as guanine by RNA polymerases and generates A>G/T>C mutations (Samuel, 2011; Harris and Dudley, 2015). It has been reported that the interferon-inducible isoform of ADAR1 may be involved in the excess of T>C and A>C changes in some measles viruses (Bass, 2002) and that APOBEC members can produce HIV-1 hypermutated proviral DNA copies (Vartanian et al., 1994). Members of the APOBEC family introduce mutations of cytidine to thymidine in single-stranded DNA during reverse transcription in the hepatitis B virus and some retroviruses (Suspène et al., 2005; Harris and Dudley, 2015). Whether the excess of C>T changes along SARS-CoV-2 genomes is a consequence of APOBEC-driven mechanisms or not has been widely discussed (Di Giorgio et al., 2020; Simmonds, 2020; Graudenzi et al., 2021; Ratcliff and Simmonds, 2021). Different studies have reported APOBEC3A-mediated mRNA editing and a 1.5 to 3-fold increment in C>T transitions with a 5’U context compared to other upstream bases within the SARS-CoV-2 genome (Sharma et al., 2015; Sharma et al., 2017). More recently, it was reported that APOBEC3A (A3A) cytidine deaminase has a pivotal role in the excess of C>T changes in the SARS-CoV-2 genome, which strongly suggest that A3A is the primary host player that shapes the mutation profile in the SARS-CoV-2 genome (Nakata et al., 2023).

Another antiviral mechanism is the one carried out by zinc finger antiviral proteins (ZAP), which are cytoplasmic proteins that target RNA viruses to protect cells from infections through the recognition of CG dinucleotides (Meagher et al., 2019). The consequences of purifying selection on synonymous mutations have been discussed in previous studies; the high mutation rate in the CG dinucleotide context in the SARS-CoV-2 genome has been interpreted as an evasion mechanism to the activity of the antiviral proteins ZAP of the host and has been reported in other coronaviruses (Woo et al., 2007; Xia, 2020; Mourier et al., 2021). Our results showed an increase in mutations in the context of the CG dinucleotide which can be interpreted as a decrease in CG dinucleotides that can be targeted by ZAP, evading degradation.

In addition, a report showed that genome-scale RNA secondary structure was significantly associated with C>T/T>C transitions in SARS-CoV-2, which could be an indication that C>T changes may be influenced by RNA structure (Simmonds and Ansari, 2021). All these mutational asymmetry mechanisms still need to be fully characterized but evidence suggests that the increase in C>T changes across the SARS-CoV-2 genome is relevant in nucleotide variability and has an important role in viral sequence diversification and evolution.

From the perspective of molecular epidemiology, we observed two general genomic patterns of variant distribution in our data: a heterogeneous pattern detected in samples from 2020 and a more homogeneous arrangement from January 2021 onwards. The genetic composition of the viral genomes showed a reduction in the number of genetic mutations that were dispersed in the whole genome towards the fixation of mutations in the ORF1a, ORF1b, S, and N regions. This reduction of genetic variation leads to the appearance and eventual dominance of the B.1.1.519 variant and the loss of other genetic variants that may have lower biological fitness.

The B.1.1.519 SARS-CoV-2 lineage was reported to harbor 20 mutations, from which 11 are nonsynonymous and four are within the spike protein. This variant was first detected in Mexico in November 2020, and in August 2021 was reported as a potential variant of interest (VOI). This lineage was derived from B.1.1.222 which was characterized by the presence of the spike mutation T478K. In addition, the B.1.1.519 lineage also presents P681H and T732A spike mutations. By May 2021 it had spread to 31 countries and was predominantly found in Mexico, followed by the USA, Canada, and Germany (Rodríguez-Maldonado et al., 2021).

The S protein is a key element of the viral-host molecular interaction. Therefore, any structural changes in this protein are crucial for the infectivity, evolution, and immunological detection of SARS-CoV-2 (Huang et al., 2020). The S aa changes could induce neutralization escape by antibodies generated by vaccines (Garcia-Beltran et al., 2021; Koyama et al., 2022). We detected the S:D614G substitution in all our samples. This mutation was first described in January 2020 and originated the B.1 lineage (Hughes et al., 2022). In May 2020, its prevalence started to rise, and by June 2020 was reported in approximately 74% of all SARS-CoV-2 published sequences (Yurkovetskiy et al., 2020). The D614G mutation is known to enhance SARS-CoV-2 infectivity, competitive fitness, transmission, and viral replication in animal models and human cells (Hou et al., 2020; Korber et al., 2020; Plante et al., 2021). This mutation emerged because of natural selection in the spike protein in the SARS-CoV-2 which caused the global pandemic (Zhang et al., 2020). Later, additional mutations appeared in several domains of the spike protein providing an increased fitness to the SARS-CoV-2, resulting in an increased threat to human health (Magazine et al., 2022). The T478K mutation had a high prevalence in Mexico and a rapid expansion (Di Giacomo et al., 2021). This mutation is located in the ACE2 interaction domain and increases its positive charge. It has been reported that this mutation enhanced RBD–ACE2 complex stabilization (Cherian et al., 2021). Other amino acid change detected in S was P681H, which has been described to reduce endosomal cathepsins dependency and to augment cell surface access (Lista et al., 2022). However, other study concluded that this mutation does not significantly affect viral cellular entry (Lubinski et al., 2022). Although the T732A mutation has been reported in Mexico, no functional studies have been done to determine its biological effects (Barona-Gómez et al., 2021; Cedro-Tanda et al., 2021; Rodríguez-Maldonado et al., 2021).

During the pandemic, SARS-CoV-2 variants have undergone extensive evolutionary transitions and ultimately achieved global dominance (Telenti et al., 2022). In México, the first case of SARS-CoV-2 was reported on 28 February 2020 (Garcés-Ayala et al., 2020) while the first patient carrying variant B.1.1.519 was detected in Mexico City on 3 November 2020. Our analysis of SARS-CoV-2 evolution in Mexico between June 2020 and February 2021 suggests a transition of variants from B.1.1.222 to B.1.1.519. The variant B.1.1.222 is characterized by the S:D614G mutation, whereas B.1.1.519 has the additional aa changes T478K, P681H, and T732A in the spike protein. In November and December 2020, we detected 27.3% (3/11) and 30% (3/10) of patients with B.1.1.222, respectively, but none of the patients from 2021 had this variant. The prevalence of B.1.1.519 increased from 18.2% (2/11) and 20% (2/10) in November and December 2020, respectively, to 82.35% (14/17) in January 2021. Although our sample is scarce, these findings are aligned with previous reports from Mexico City where B.1.1.222 prevalence decreased in the later months of 2020, while B.1.1.519 increased in the first months of 2021, corresponding to the second COVID-19 wave in Mexico (Cedro-Tanda et al., 2021; Rodríguez-Maldonado et al., 2021; Taboada et al., 2021).

Our phylogenetic analysis showed that the 50 genomes of SARS-CoV-2 analyzed were grouped in three main clusters, according to NextClade. The B.1.1.519 and B.1.1.222 variants were grouped in independent clades, with B.1.1.222 derived from the 20A clade and B.1.1.519 from the 20B clade. These variants shared a most recent common ancestor with the mutation D614G in the spike protein, however, B.1.1.519 acquired the three additional aa changes previously described.

Pathogenic viruses have shown genetic evolution inside the host. Some reports have described intrapatient heterogeneity in Ebola, Middle East respiratory syndrome coronavirus, and SARS-CoV-2, characterized by the coexistence of several viral subclones (Ni et al., 2016; Park et al., 2016; Wang et al., 2021). In our dataset, the mean allelic fraction was 98.29% (range 72.3-100%; data not shown), and mutation sequencing depth mean was (15,867X; range 14-150,328; data not shown). In addition, with this analysis every patient only presented a single group of mutations, an indication of one single viral variant per patient. Therefore, even by sequencing at a high depth there was no clear evidence of subclonal variants, which suggests that all viruses might be clonal in the patients. However, since our samples were obtained from critical patients that have a long disease course, we cannot discard the possibility of SARS-CoV-2 evolving withing the host, especially in the patients from the first wave (2020), where there was significant variability in the circulating viral variants.

We are aware that our study has some limitations that we believe do not affect the quality or the results obtained, which include (i) the lack of clinical variables and therefore their association with the disease, (ii) the sample size, which may appear to be small when compared with global epidemiological works but is similar to other SARS-CoV-2 genomic studies (Taboada et al., 2020; Elizondo et al., 2021; Yavarian et al., 2022), and (iii) the temporality of the samples.

The evolutionary behavior of SARS-CoV-2 throughout the pandemic has challenged vaccine development and has complicated the landscape for current treatments due to the adaptive mechanisms of the virus in response to the host immune system. This work on the mutational profile of the SARS-CoV-2 genome may contribute to a better understanding of the evolutionary dynamics and selective pressures acting on the SARS-CoV-2 genome, the prediction of more accurate variants of clinical significance, and a better understanding of the molecular mechanisms driving SARS-CoV-2 evolution to improve vaccine and drug development.

## Data availability statement

The data presented in the study are deposited in the Sequence Read Archive repository, accession number PRJNA967733.

## Ethics statement

The studies involving human participants were reviewed and approved by National Institute of Respiratory diseases Ethics Committee. The patients/participants provided their written informed consent to participate in this study.

## Author contributions

Conception, AD-M, JZ, FA-M, and FV-P. Patient recruitment, sampling, database, JB, LJ-A, NR-Z, GR-M, AC-L, IP, LL, BC-G, GC, CG, RS, and FA-M. Experimental analysis, AD-M, CD, JB, TC-J, CG. Data analysis and visualization, AD-M, HM-G, MD, and FV-P. Manuscript writing and review, AD-M, CD, HM-G, MD, JB, TC-J, JO, MR, LV, IP, JZ, FA-M, and FV-P. Resources, LV, JZ, FA-M, and FV-P. Funding acquisition, FA-M. All authors contributed to the article and approved the submitted version.
